# Protective Effect of Intravenous High Molecular Weight Polyethylene Glycol on Fatty Liver Preservation

**DOI:** 10.1155/2015/794287

**Published:** 2015-10-12

**Authors:** Mohamed Bejaoui, Eirini Pantazi, Emma Folch-Puy, Arnau Panisello, María Calvo, Gianfranco Pasut, Antoni Rimola, Miquel Navasa, René Adam, Joan Roselló-Catafau

**Affiliations:** ^1^Experimental Pathology Department, Institute of Biomedical Research of Barcelona (IIBB-CSIC), 08036 Barcelona, Catalonia, Spain; ^2^Serveis Cientifico-Tècnics, Universitat de Barcelona, 08036 Barcelona, Catalonia, Spain; ^3^Pharmaceutical and Pharmacological Sciences Department, University of Padova, 35122 Padova, Italy; ^4^Liver Unit, Hospital Clinic Barcelona, IDIBAPS, University of Barcelona, 08036 Barcelona, Catalonia, Spain; ^5^Centro de Investigación Biomédica en Red de Enfermedades Hepáticas y Digestivas (CIBEREHD), Barcelona, Catalonia, Spain; ^6^Centre Hepato-Biliaire, AP-P-HP Hôpital Paul Brousse, Inserm U776, Université Paris Sud, Villejuif, 75008 Paris, France

## Abstract

Ischemia reperfusion injury (IRI) leads to significant tissue damage in liver surgery. Polyethylene glycols (PEGs) are water soluble nontoxic polymers that have proved their effectiveness against IRI. The objective of our study was to investigate the potential protective effects of intravenous administration of a high molecular weight PEG of 35 kDa (PEG 35) in steatotic livers subjected to cold ischemia reperfusion. In this study, we used isolated perfused rat liver model to assess the effects of PEG 35 intravenous administration after prolonged cold ischemia (24 h, 4°C) and after reperfusion (2 h, 37°C). Liver injury was measured by transaminases levels and mitochondrial damage was determined by confocal microscopy assessing mitochondrial polarization (after cold storage) and by measuring glutamate dehydrogenase activity (after reperfusion). Also, cell signaling pathways involved in the physiopathology of IRI were assessed by western blot technique. Our results show that intravenous administration of PEG 35 at 10 mg/kg ameliorated liver injury and protected the mitochondria. Moreover, PEG 35 administration induced a significant phosphorylation of prosurvival protein kinase B (Akt) and activation of cytoprotective factors e-NOS and AMPK. In conclusion, intravenous PEG 35 efficiently protects steatotic livers exposed to cold IRI.

## 1. Introduction

Organ preservation is a fundamental requirement in organ transplantation; it preserves the viability of the organ during its transport from the donor to the recipient so that the graft can maintain its function after transplantation [1]. Besides advances in organ preservation, the presence of steatosis remains a limiting factor for the suitable preservation of liver grafts, as steatotic livers are particularly vulnerable to hepatic ischemia reperfusion injury (IRI) [2]. Their use is accompanied by increased risk of primary failure and lowered success of liver transplantation [3]. Currently, the increasing needs of transplantation as well as the scarce of donors pool have obliged the physicians to take advantage of suboptimal liver grafts, as steatotic ones [4]. For this reason, there is an urgent need to explore new strategies that provide a more efficient preservation of steatotic liver grafts. Minimizing the deleterious effects of hypothermia could decrease the reperfusion injury and, consequently, assure an increased rate of graft survival after transplantation.

Polyethylene glycols (PEGs) are water soluble nontoxic polymers that have been employed in many biomedical applications such as gastrointestinal disorders and drugs pegylation [5, 6]. Besides their usefulness as oncotic agents in preservation solutions [7, 8], it has been shown that PEGs molecules protect against cold injury and ischemic damage. Indeed, PEG used as cryoprotectant in supercooling technique was necessary to achieve successful liver transplantation [9]. Moreover, PEG suppressed hypothermic-induced cell swelling in hepatocyte preservation [10] and protected primary hepatocyte during supercooling preservation [11]. Also, PEG protected cardiac myocytes from hypoxia and reoxygenation-induced cell death [12], decreased oxidative stress [13], and protected injured mitochondria [14].

With this in mind, we hypothesized whether intravenous administration of PEG in the rat prior to organ procurement could protect fatty liver graft against hypothermic and hypoxic damage occurring during preservation and the subsequent reperfusion injury. Our results demonstrated that PEG 35 prevented the deleterious effects of cold IRI when administered intravenously in obese rats.

## 2. Materials and Methods

### 2.1. Animals

Male homozygous obese Zucker rats, aged 9 to 10 weeks, were purchased from Charles River (France) and housed at 22°C with free access to water and standard chow. All experiments were approved by the Ethics Committees for Animal Experimentation (CEEA, Directive 697/14), University of Barcelona, and were conducted according to European Union regulations for animal experiments (Directive 86/609 CEE).

### 2.2. Liver Procurement and* Ex Vivo* Perfusion

All procedures were performed under isoflurane anesthesia inhalation. After laparotomy, the common bile duct was cannulated and livers were flushed with 40 mL of chilled UW preservation solution (4°C) by the mean of catheter insertion into the aorta. After cooling, a second catheter was inserted into the portal vein to complete liver rinsing with further 10 mL of UW solution. The whole liver was then excised and preserved at 4°C for 24 h in the same solution. This procedure implicates the death of the animal under isoflurane anesthesia, and thus the application of analgesia or euthanasia was unnecessary. After 24 h of cold preservation, steatotic livers were removed from preserved solution and flushed at room temperature with 20 mL of Ringer Lactate solution to eliminate the metabolite waste accumulated during liver storage. Then, livers were perfused at 37°C via the portal vein in a closed and controlled pressure circuit. Time point 0 was considered when the portal catheter was satisfactorily connected to the circuit. During the first 15 minutes of perfusion (initial equilibration period), the flow was progressively increased in order to stabilize the portal pressure at 12 mmHg (Pression Monitor BP-1; Pression Instruments, Sarasota, FL). The flow was controlled by a peristaltic pump (Minipuls 3; Gilson, France). The reperfusion liquid (150 mL for each perfusion) consisted of a cell culture medium (William's medium E; BioWhittaker, Barcelona, Spain) with a Krebs-Henseleit-like electrolyte composition enriched with 5% albumin as oncotic supply. The medium was continuously gassed with 95% O_2_ and 5% CO2 gas mixture and subsequently passed through a heat exchanger (37°C) and a bubble trap prior to entering the liver. After 120 minutes of normothermic reperfusion, the effluent perfusion fluid was collected for biochemical determinations and hepatic tissues were sampled and stored at −80°C for further analysis.

### 2.3. Drug Treatment

PEG 35 was kindly provided by IGL-1 Company. PEG 35 was dissolved in physiological saline (5 g/L) and administrated 10 min before liver procurement by intravenous bolus through the penile vein at the concentration of 10 mg/kg.

For confocal microscopy study with PEG-FITC, PEG 35 was fused with fluorescein as previously described by Mero et al. [15].

### 2.4. Experimental Groups

**Scheme 1:**
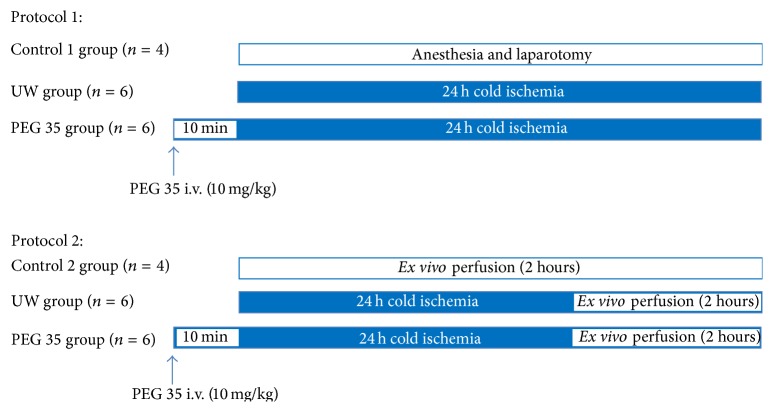
Experimental designs to investigate PEG 35 effects on steatotic livers after cold ischemia at 4°C (Protocol 1) and after cold storage followed by normothermic* ex vivo* reperfusion (Protocol 2).

All animals were randomly distributed into different experimental groups, as indicated below (Scheme 1).

*Protocol 1: Effect of PEG 35 in Fatty Livers after Cold Storage.* In order to study the effects of PEG 35 administration in cold preservation, rats were randomly divided into the following groups:

(1) Group  1: Control 1 (Ctr 1) (*n* = 4): control livers were flushed via the portal vein with Ringer's lactate solution immediately after laparotomy. Then liver samples were collected for posterior analysis.
(2) Group  2 (*n* = 6): UW: steatotic livers were preserved for 24 hours in UW solution at 4°C. Then, livers were flushed with Ringer's solution and the effluent liquid was collected for further biochemical determinations. Liver tissue was stored at −80°C.
(3) Group  3 (*n* = 6): PEG 35: livers were pretreated with PEG 35 intravenously at 10 mg/kg 10 min before liver procurement and then preserved for 24 hours in UW solution as in group 2.

*Protocol 2: Effect of PEG 35 in Fatty Livers after 24 h of Cold Storage and 2 h of Normothermic Reperfusion.* To examine the effect of PEG 35 in liver injury after normothermic perfusion, fatty livers were randomized in the following groups:

(1) Control group (Ctr 2) (*n* = 4): after procurement, steatotic livers were* ex vivo* perfused for 2 h at 37°C as described above, without prior cold storage.
(2) UW group (*n* = 6): fatty livers were preserved in UW preservation solution for 24 hours at 4°C and then subjected to 2 h of normothermic reperfusion at 37°C.
(3) PEG 35 group (*n* = 6): Zucker Ob rats were pretreated with intravenous administration of PEG 35 at 10 mg/kg, 10 min before liver procurement. Then, livers were preserved for 24 h in UW solution and finally* ex vivo* perfused for 2 hours at 37°C.

### 2.5. Liver Injury: Transaminases Assay

Hepatic injury was assessed in terms of alanine aminotransferase (ALT) and aspartate aminotransferase (AST) levels with commercial kits from RAL (Barcelona, Spain). Briefly, 100 *μ*L of effluent washout liquid or perfusate was added to 1 mL of the substrate provided by the commercial kit and then transaminases activity was measured at 340 nm with a UV spectrometer and calculated following the supplier's instructions. Results were normalized using a commercial calibrator (Biocal, RAL, Barcelona, Spain).

### 2.6. Mitochondrial Damage

#### 2.6.1. Glutamate Dehydrogenase Activity

Glutamate dehydrogenase (GLDH) is a mitochondrial enzyme present predominantly in liver and contributes to the oxidative deamination of glutamate. Measurable increases in serum levels are indicative of mitochondrial damage. Serum concentrations of GLDH were determined using a commercial kit (GLDH, Randox laboratories Ltd., Crumlin, UK) by quantifying the decrease in absorbance at 340 nm according to the manufacturer's protocol.

#### 2.6.2. Confocal Microscopy

After 24 h of hypothermic preservation, fatty livers pretreated with PEG conjugated to FITC (PEG-FITC) or saline were washed out via the portal vein with 20 mL of Ringer lactate solution containing fluorescent dyes. The fluorescent dyes were diluted in the washout liquid and injected to the preserved fatty liver at the following final concentrations: Hoechst 33342 trihydrochloride (12 mg/kg body weight, Invitrogen, H3570) for DNA-nuclei staining and rhodamine 123 (0.11 mg/kg body weight, Sigma, R8004) for mitochondrial membrane potential staining. Fatty livers were then carefully sectioned (0.5 cm^3^ fragments) and the internal side of the liver was exposed on the glass coverslip mounted on the stage of a Leica TCS SP5 resonant scan multiphoton confocal microscope (Leica Microsystems Heidelberg GmbH) equipped with a HCX IR APO L 25x water immersion objective (Numerical Aperture 0.95), scanner at 400 lines/s, and a near infrared Titanium:Saphire laser (MaiTai, SpectraPhysics) for two-photon excitation running at 800 nm. Images were acquired with resonant scan at 8000 lines/second. Two-photon excitation was performed at 800 nm and emission of the different fluorescent dyes was captured at the following wavelength ranges: PEG-FITC (400–550 nm), Hoechst 33342 (400–470 nm), and rhodamine 123 (500–550 nm).

### 2.7. Vascular Resistance

Vascular resistance was defined as the ratio of portal venous pressure which was maintained at 12 mmHg during the reperfusion to flow rate and expressed in mmHg/min per gram of liver/mL. Perfusion flow rate was assessed continuously throughout the reperfusion period and expressed as mL/min per gram of liver.

### 2.8. Western Blotting Technique

Liver tissue was homogenized in HEPES buffer and proteins were separated by SDS-PAGE and transferred to PVDF membranes. Then, membranes were immunoblotted over night at 4°C using the following antibodies: anti-p-AMPK*α* (Thr172, #2535), anti-AMPK*α* (#2603), anti-p-SAPK/JNK (Thr183/Tyr185), anti-p-p38 MAP kinase (Thr180/Tyr182, #9211), and anti-p-p44/42 MAPK (Erk1/2, Thr202/Tyr204, #9101); the above antibodies were all purchased from Cell Signaling (Danvers, MA); anti-eNOS (610296) was purshased form Transduction Laboratories (Lexington KY) and anti-b-actin (A5316) was purshased from Sigma Chemical (St. Louis, MO, USA). After washing, bound antibody was detected after incubation for 1 h at room temperature with the corresponding secondary antibody linked to horseradish peroxidase. Bound complexes were detected and quantified by scanning densitometry.

### 2.9. Statistical Analysis

Statistical analysis was performed with GraphPad Prism version 4.02 for Windows (GraphPad Software). Quantitative data are reported as mean ± SEM and statistical comparison was performed with analysis of variance, followed by Tukey tests. An associated probability of *p* < 0.05 was considered to be significant.

## 3. Results

### 3.1. Effect of Intravenous PEG 35 on Cold Storage of Steatotic Rat Livers

**Figure 1:**
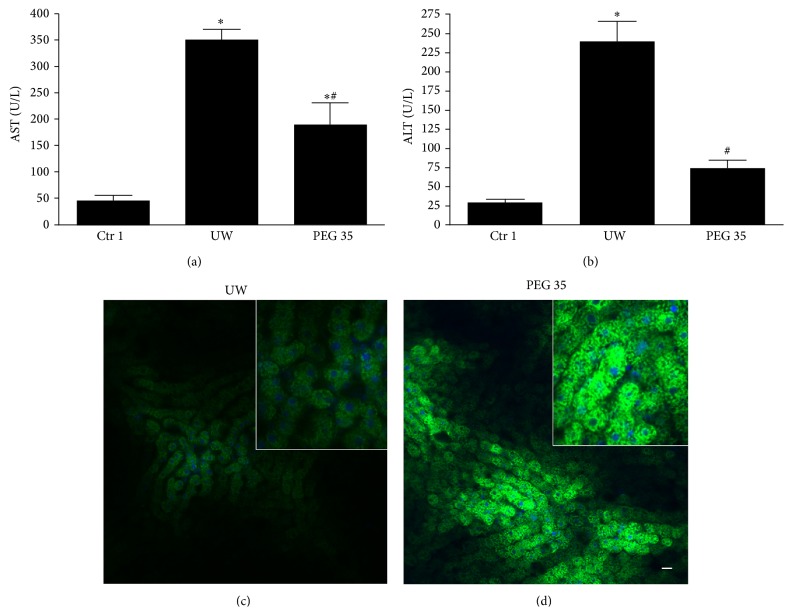
Hepatic and mitochondrial injuries after cold ischemia. PEG 35 administration decreases aspartate aminotransferase (AST) (a) and alanine aminotransferase (ALT) levels (b) after 24 h of cold storage. Confocal microscopy of mitochondrial membrane potential stained with rhodamine 123 (green) after cold storage: mitochondrial depolarization occurs after preservation (c); however, in rats pretreated with PEG 35 conjugated to FITC, we observed bright punctate fluorescence showing polarized mitochondria (d). No PEG fluorescence has been detected in liver sinusoids, neither in hepatocytes nor bound to cell membrane (d). Ctr 1: anaesthesia and laparotomy; UW: livers preserved in UW preservation solution for 24 hours at 4°C; PEG 35: Zucker obese rats treated intravenously with PEG 35 at 10 mg/kg and steatotic livers were then subjected to 24 h cold ischemia. Data represent mean ± SEM. ^*∗*^ *p* < 0.05 versus Ctr 1; ^#^ *p* < 0.05 versus UW.

To investigate the protective effect of intravenous PEG 35 treatment on liver preservation, we measured transaminases levels in the effluent of washout liquid after 24 h of cold storage. As shown in Figures 1(a) and 1(b), liver preservation resulted in increased AST/ALT levels versus control group and the intravenous administration of PEG 35 at 10 mg/kg decreased significantly transaminases release indicating substantially less hepatocellular damage. Also, we explored mitochondrial polarization after fatty liver preservation using rhodamine 123 vital dye. In livers pretreated with PEG 35, we observed bright punctate fluorescence standing for the cells with polarized mitochondria. By contrast, in nontreated livers, we observed a cloudy diffuse cytosolic fluorescence standing for cells with depolarized mitochondria (Figures 1(c) and 1(d)). Moreover, our result shows that when obese rats were treated with PEG-FITC, no significant PEG fluorescence was detected in liver sinusoids neither into hepatocytes or other liver cells nor bound to cell membrane (Figure 1(d)).

### 3.2. Effect of Intravenous PEG 35 Administration on Fatty Liver Injury after Ischemia Reperfusion

**Figure 2:**
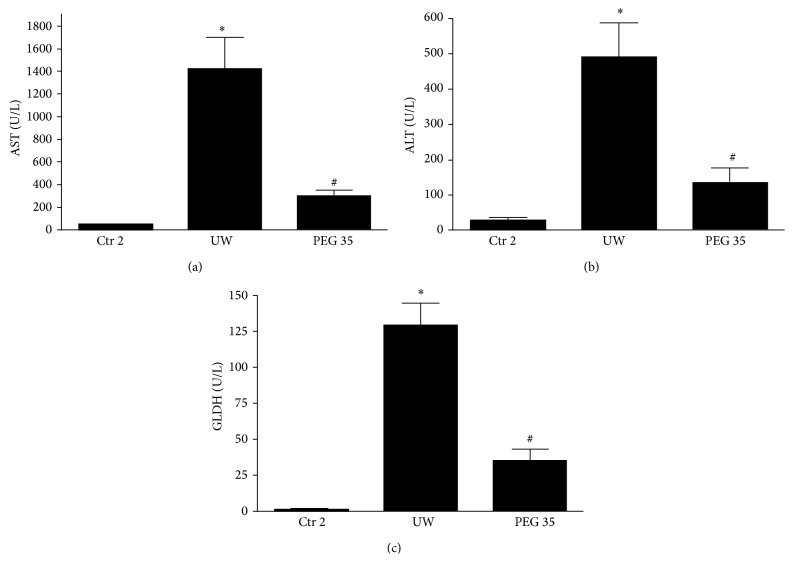
Hepatic and mitochondrial injuries after cold ischemia and reperfusion. PEG 35 administration decreases aspartate aminotransferase (AST) (a), alanine aminotransferase (ALT) (b), and glutamate dehydrogenase (GLDH) levels after 2 hours of* ex vivo* perfusion. Ctr 2: liver procurement and* ex vivo* perfusion; UW: livers preserved in UW preservation solution for 24 h at 4°C and then subjected to 2 h of normothermic* ex vivo* perfusion; PEG 35: rats treated intravenously with PEG 35 (10 mg/kg) and then subjected to 24 h cold ischemia followed by 2 h of normothermic* ex vivo* perfusion. Data represent mean ± SEM. ^*∗*^ *p* < 0.05 versus Ctr 2; ^#^ *p* < 0.05 versus UW.

In the following, we evaluated the reperfusion injury after 2 hours of* ex vivo* perfusion at 37°C (Protocol 2). We observed a significant decrease in transaminases levels in the perfusate from rats pretreated with PEG 35 when compared to the untreated ones (Figures 2(a) and 2(b)). Moreover, the evaluation of mitochondrial damage, measured by GLDH activity, showed significant decreases when rats were pretreated with PEG 35 (Figure 2(c)).

**Figure 3:**
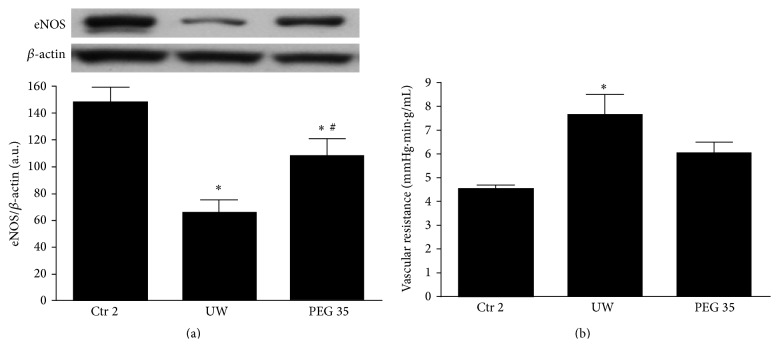
Effects of PEG 35 on eNOS activation and vascular resistance. PEG 35 pretreatment activates eNOS and decreases vascular resistance: densitometric analysis of eNOS/*β*-actin (a) and vascular resistance (b) after 120 min of normothermic reperfusion. Ctr 2: liver procurement and* ex vivo* perfusion; UW: livers preserved in UW preservation solution for 24 h at 4°C and then subjected to 2 h of normothermic* ex vivo* perfusion; PEG 35: rats treated intravenously with PEG 35 (10 mg/kg) and then subjected to 24 h cold ischemia followed by 2 h of normothermic* ex vivo* perfusion. Data represent mean ± SEM. ^*∗*^ *p* < 0.05 versus Ctr 2; ^#^ *p* < 0.05 versus UW.

Steatotic livers present fat accumulation in the cytoplasm of the hepatocytes which causes disturbance of the sinusoidal flow during reperfusion [2, 16]. Given that, we explored vascular resistance and endothelial nitric oxide synthase (eNOS) activation after reperfusion.  Figure 3(a) shows that eNOS protein expression decreased after 2 h of* ex vivo* perfusion in UW compared to control group. In contrast, pretreatment with PEG 35 clearly induced eNOS expression which was concomitant with decreased vascular resistance (Figure 3(b)).

**Figure 4:**
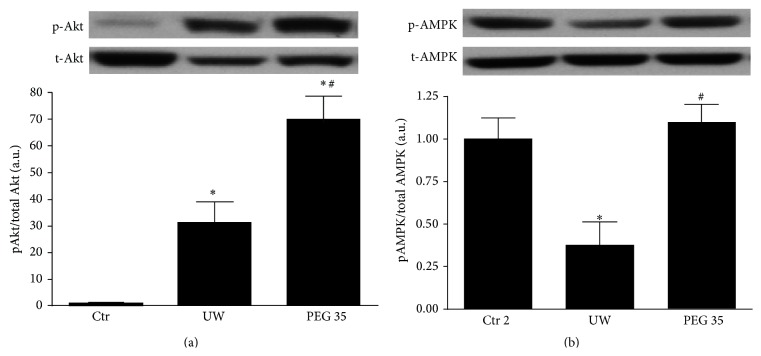
Effects of PEG 35 on Akt and AMPK. PEG 35 administration activates Akt and AMPK: densitometric analysis of phosphorylated Akt/total Akt (a) and phosphorylated AMPK/total AMPK (b). Ctr 2: liver procurement and* ex vivo* perfusion; UW: livers preserved in UW preservation solution for 24 h at 4°C and then subjected to 2 h of normothermic* ex vivo* perfusion; PEG 35: rats treated intravenously with PEG 35 (10 mg/kg) and then subjected to 24 h cold ischemia followed by 2 h of normothermic* ex vivo* perfusion. Data represent mean ± SEM. ^*∗*^ *p* < 0.05 versus Ctr 2; ^#^ *p* < 0.05 versus UW.

Next, we explored whether the hepatoprotective effect of PEG 35 could be attributed to well-known cell signaling pathways associated with IRI such as adenosine monophosphate activated protein kinase (AMPK) and protein kinase B (Akt). As shown in Figure 4(a), liver preservation followed by 2 hours of* ex vivo* perfusion promoted Akt phosphorylation, which was further enhanced when rats were pretreated with PEG 35. Regarding AMPK, PEG 35 administration prior to liver procurement induced a significant activation in AMPK in comparison to non-PEG 35-treated rats (Figure 4(b)).

**Figure 5:**
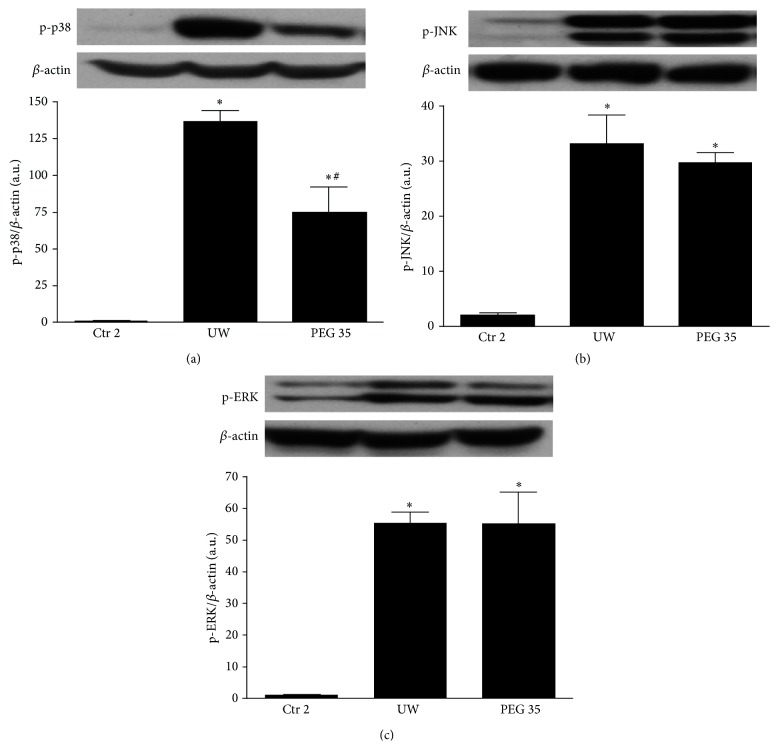
Effect of PEG 35 on MAPKs. PEG 35 reduces p38-MAPK activation whereas it has no effect on JNK and ERK phosphorylation. Densitometric analysis of phosphorylated p38/*β*-actin (a), phosphorylated JNK/*β*-actin (b), and phosphorylated ERK/*β*-actin (c). Ctr 2: liver procurement and* ex vivo* perfusion; UW: livers preserved in UW preservation solution for 24 h at 4°C and then subjected to 2 h of normothermic* ex vivo* perfusion; PEG 35: rats treated intravenously with PEG 35 (10 mg/kg) and then subjected to 24 h cold ischemia followed by 2 h of normothermic* ex vivo* perfusion. Data represent mean ± SEM. ^*∗*^ *p* < 0.05 versus Ctr 2; ^#^ *p* < 0.05 versus UW.

It is well known that mitogen activated protein kinase (MAPK) signaling pathway regulates inflammation and cell survival during IRI [17, 18]. We therefore assessed the possible involvement of MAPK regulation in the protective effect of PEG 35. As indicated in Figure 5, all MAP kinases (p-p38, p-JNK, and p-Erk) levels were increased at 2 h reperfusion. A significant reduction in p-p38 activation was evident after PEG 35 treatment (Figure 5(a)). On the contrary, no changes for JNK and Erk activity were found (Figures 5(b) and 5(c), resp.).

## 4. Discussion

The beneficial effects of PEG in tissue injury are well documented [5, 12–14, 19]. However, because PEG molecules are not absorbed in the gastrointestinal tracts, their use against ischemic damage was limited to their addition to preservation solutions as oncotic agents. The present study was thus designed to investigate if the intravenous PEG 35 administration by a unique and nontoxic dose of 10 mg/kg could protect steatotic liver grafts against the deleterious effects of cold storage and the subsequent reperfusion. Our data demonstrated that pretreatment of rats with PEG 35 lessened liver injury associated with ischemia reperfusion.

In our study, we have used the isolated perfused rat liver (IPRL) model, a widely used and appreciated method to assess cellular injury and liver function in an isolated setting. In comparison to other* in vitro* models, the IPRL-model does have considerable advantages, such as the use of the entire intact organ instead of only single cells or several layers of cells (i.e., isolated hepatocytes or the liver slice model) and an intact cellular architecture. Furthermore, the use of an acellular perfusion solution (Krebs solution) prevents alloreactivity and permits a conclusive focus on IRI effects. Regarding liver transplantation, IPRL model presents the advantages of minimizing the use of laboratory animals as well as the suppression of the immunological reactions and the influences of other organs occurring during transplantation. The disadvantage of the IPRL-model is the duration of reperfusion, which is limited to 90–120 minutes and the fact that it remains an* in vitro* tool that merely simulates the initial phase after liver transplantation. In this sense IPRL model could be considered as a pre-screening model before liver transplantation especially in ischemia reperfusion research [20, 21].

In contrast to the current pharmacological strategies used against IRI, PEG administration presents the advantages of being a multitarget strategy. In fact, IRI is a multifactorial disease including oxidative stress, inflammation, proteasome activation, endoplasmic reticulum stress, mitochondrial damage, and cytoskeleton alterations which lead to cell death and organ dysfunction [22–24]. PEG has been associated with the majority of these events as it has been shown that PEG reduces reactive oxygen species, prevents cell death, maintains mitochondrial integrity, and reduces inflammation and endoplasmic reticulum stress [12, 14, 19, 25, 26].

The half-life and biodistribution of the polymer and consequently its activity mainly depend on its molecular weight. Based on our experience in organ preservation, we used PEG with a molecular weight of 35 kDa. Indeed, we have previously demonstrated that PEG 35 addition to washout solution protected the liver against reperfusion injuries [27]. Moreover, PEG 35 addition to IGL-1 preservation solution protects kidney and liver grafts against ischemic damage [7, 8, 28, 29]. PEG with a molecular weight of 20 kDa has also been used as an additive to HTK and SCOT preservation solutions and was associated with protective effects against IRI in pancreas [30], kidney [8], intestine [26], and liver grafts [31]. In addition, PEG20 has been shown to protect against cardiomyocyte apoptosis induced by hypoxia [12]. However, PEG 35 was more effective than PEG20 in protecting porcine proximal tubular epithelial cell line against cold storage at the same doses used [32].

Mitochondrial protection is essential for graft survival after transplantation [33]. Thus, we further explored mitochondrial depolarization after cold preservation and we evidenced that PEG 35 prevented fatty liver mitochondria depolarization after prolonged cold ischemia. Also, mitochondrial injury was lessened after liver reperfusion as indicated by the decrease in GLDH release. These results are in accordance with previous published data showing that PEG 2 kDa improved mitochondrial function* in vitro* and* in vivo* after acute spinal cord injury [25]. Moreover, PEG of 4 kDa inhibited mitochondrial pore transition (MPT) and cytochrome C release in rat liver mitochondria [34]. Also, PEG (1.5 and 2 kDa) was able to cross the cytoplasmic membrane and directly interact with neuronal mitochondria to preserve its structure and restore function [14]. Interestingly, PEG with higher molecular weight (4 kDa) failed to exert significant improvement in neuronal injured mitochondria indicating that PEG-mediated mitochondrial protection is dependent on the size of PEG [14]. In our study, we did not detect any PEG fluorescence after cold storage in liver sinusoids, neither in hepatocytes nor bound to cell membrane. In this sense, the mechanism by which PEG 35 decreases mitochondrial damage and exerts its protective effects needs more profound investigation.

Our results show that PEG 35 activated eNOS, the enzyme responsible for nitric oxide (NO) generation, and consequently decreased vascular resistance. This could also explain the protective mechanism of PEG toward mitochondria as it has been showed that NO protects rat hepatocytes against reperfusion injury through the inhibition of MPT [35]. Previous study from Bertuglia et al. has shown that PEG 15–20 kDa reduced vasoconstriction and the altered capillary perfusion after ischemia reperfusion [36]. However, in that case, the decreased vascular resistance of PEG were not mediated by eNOS activation [36].

In order to explore whether the beneficial effects of PEG 35 are associated with other well-known cell signaling pathways involved in IRI, we further evaluated the activation of AMPK and Akt and the regulation of MAPKs. AMPK is a metabolic fuel gauge and energy regulator activated during ischemia in order to induce an energy-saving state preventing thus the lactate accumulation and cell death [37–39]. Here, we showed that PEG 35 enhanced AMPK levels after reperfusion, which could contribute to assuring energy levels sufficient to cell survival. Another cytoprotective marker is Akt, a serine-threonine protein kinase that is linked to cell survival during reperfusion [40–42]. Data reported here revealed that PEG 35 increased Akt levels, as similarly observed with PEG 20 in cardiac myocyte submitted to IRI [12]. Regarding MAPKs signalling, we observed that PEG 35 was capable of preventing p38 activation, while no changes were found on JNK and ERK pathways. The data reported here are consistent with previously reported works showing that the inhibition of p38 prevented preservation-induced graft injury and improved the outcome of liver transplantation [43–45]. Other studies as well reported that PEG 35 decreased p38 activation while it activated JNK in cold stored porcine proximal tubular cell line [32].

The rationale of PEG 35 intravenous administration was to induce a pharmacological preconditioning against the subsequent cold storage and reperfusion injury. PEG presents the advantages of being safe and multifactorial agent and may constitute a novel strategy to increase liver graft preservation. This could be relevant in clinical situation of brain-dead donors or steatotic livers, both being risk factors in liver transplantation. Until now, PEG has been used clinically for ischemia reperfusion purpose as additive to preservation solution due to its oncotic properties. In this study, we used UW solution which contains hydroxyethyl starch as an oncotic support in order to demonstrate that the protective mechanisms of PEG are not only related to its oncotic effect, but also to other properties such as the preservation of mitochondria and the induction of protective cell signaling pathway (eNOS, Akt, and AMPK). In a previous study we have shown that PEG addition to rinse solution protected preserved liver against the subsequent reperfusion injury (PEG postconditioning). Interestingly, when liver grafts were preserved in IGL-1 solution which contains PEG 35, the rinse solution does not show any additional protective effect [46]. In this sense, PEG pre- and postconditioning would be considered as a safe and protective strategy applicable to all preservation solutions.

## 5. Conclusions

PEG 35 represents a potential pharmacological agent for preventing the deleterious effects of cold IRI and may constitute a novel clinical strategy to increase liver graft preservation, especially for “marginal” organs.

## References

[1] McLaren A. J., Friend P. J. (2003). Trends in organ preservation. *Transplant International*.

[2] Peralta C., Roselló-Catafau J. (2004). The future of fatty livers. *Journal of Hepatology*.

[3] Chu M. J., Dare A. J., Phillips A. R., Bartlett A. S. (2015). Donor hepatic steatosis and outcome after liver transplantation: a systematic review. *Journal of Gastrointestinal Surgery*.

[4] Dutkowski P., Schlegel A., Slankamenac K. (2012). The use of fatty liver grafts in modern allocation systems: risk assessment by the balance of risk (BAR) score. *Annals of surgery*.

[5] Parnaud G., Taché S., Peiffer G., Corpet D. E. (1999). Polyethylene-glycol suppresses colon cancer and causes dose-dependent regression of azoxymethane-induced aberrant crypt foci in rats. *Cancer Research*.

[6] Pasut G., Veronese F. M. (2012). State of the art in PEGylation: the great versatility achieved after forty years of research. *Journal of Controlled Release*.

[7] Ben Mosbah I., Saidane D., Peralta C., Roselló-Catafau J., Ben Abdennebi H. (2005). Efficacy of polyethylene glycols in University of Wisconsin preservation solutions: a study of isolated perfused rat liver. *Transplantation Proceedings*.

[8] Thuillier R., Renard C., Rogel-Gaillard C. (2011). Effect of polyethylene glycol-based preservation solutions on graft injury in experimental kidney transplantation. *British Journal of Surgery*.

[9] Bruinsma B. G., Berendsen T. A., Izamis M. L., Yeh H., Yarmush M. L., Uygun K. (2015). Supercooling preservation and transplantation of the rat liver. *Nature Protocols*.

[10] Mack J. E., Kerr J. A., Vreugdenhil P. K., Belzer F. O., Southard J. H. (1991). Effect of polyethylene glycol on lipid peroxidation in cold-stored rat hepatocytes. *Cryobiology*.

[11] Puts C. F., Berendsen T. A., Bruinsma B. G. (2015). Polyethylene glycol protects primary hepatocytes during supercooling preservation. *Cryobiology*.

[12] Malhotra R., Valuckaite V., Staron M. L. (2011). High-molecular-weight polyethylene glycol protects cardiac myocytes from hypoxia- and reoxygenation-induced cell death and preserves ventricular function. *The American Journal of Physiology—Heart and Circulatory Physiology*.

[13] Luo J., Borgens R., Shi R. (2002). Polyethylene glycol immediately repairs neuronal membranes and inhibits free radical production after acute spinal cord injury. *Journal of Neurochemistry*.

[14] Chen H., Quick E., Leung G. (2009). Polyethylene glycol protects injured neuronal mitochondria. *Pathobiology*.

[15] Mero A., Clementi C., Veronese F. M., Pasut G. (2011). Covalent conjugation of poly(ethylene glycol) to proteins and peptides: strategies and methods. *Methods in Molecular Biology*.

[16] Ramalho F. S., Fernandez-Monteiro I., Rosello-Catafau J., Peralta C. (2006). Hepatic microcirculatory failure. *Acta Cirurgica Brasileira*.

[17] King L. A., Toledo A. H., Rivera-Chavez F. A., Toledo-Pereyra L. H. (2009). Role of p38 and JNK in liver ischemia and reperfusion. *Journal of Hepato-Biliary-Pancreatic Surgery*.

[18] Cowan K. J., Storey K. B. (2003). Mitogen-activated protein kinases: new signaling pathways functioning in cellular responses to environmental stress. *Journal of Experimental Biology*.

[19] Shi R. (2013). Polyethylene glycol repairs membrane damage and enhances functional recovery: a tissue engineering approach to spinal cord injury. *Neuroscience Bulletin*.

[20] Hart N. A. T., van der Plaats A., Moers C. (2006). Development of the isolated dual perfused rat liver model as an improved reperfusion model for transplantation research. *The International Journal of Artificial Organs*.

[21] Bessems M., 'T Hart N. A., Tolba R. (2006). The isolated perfused rat liver: standardization of a time-honoured model. *Laboratory Animals*.

[22] Bejaoui M., Zaouali M. A., Folch-Puy E. (2014). Bortezomib enhances fatty liver preservation in Institut George Lopez-1 solution through adenosine monophosphate activated protein kinase and Akt/mTOR pathways. *Journal of Pharmacy and Pharmacology*.

[23] Zaouali M. A., Ben Mosbah I., Boncompagni E. (2010). Hypoxia inducible factor-1alpha accumulation in steatotic liver preservation: role of nitric oxide. *World Journal of Gastroenterology*.

[24] Shinohara H., Tanaka A., Fujimoto T. (1996). Disorganization of microtubular network in postischemic liver dysfunction: its functional and morphological changes. *Biochimica et Biophysica Acta—Molecular Basis of Disease*.

[25] Luo J., Borgens R., Shi R. (2004). Polyethylene glycol improves function and reduces oxidative stress in synaptosomal preparations following spinal cord injury. *Journal of Neurotrauma*.

[26] Valuckaite V., Seal J., Zaborina O., Tretiakova M., Testa G., Alverdy J. C. (2013). High molecular weight polyethylene glycol (PEG 15-20) maintains mucosal microbial barrier function during intestinal graft preservation. *Journal of Surgical Research*.

[27] Zaouali M. A., Bejaoui M., Calvo M. (2014). Polyethylene glycol rinse solution: an effective way to prevent ischemia-reperfusion injury. *World Journal of Gastroenterology*.

[28] Ben Abdennebi H., Steghens J.-P., Hadj-Aïssa A. (2002). A preservation solution with polyethylene glycol and calcium: a possible multiorgan liquid. *Transplant International*.

[29] Zaouali M. A., Ben Abdennebi H., Padrissa-Altés S., Alfany-Fernandez I., Rimola A., Roselló-Catafau J. (2011). How Institut Georges Lopez preservation solution protects nonsteatotic and steatotic livers against ischemia-reperfusion injury. *Transplantation Proceedings*.

[30] Squifflet J.-P., Ledinh H., De Roover A., Meurisse M. (2011). Pancreas preservation for pancreas and islet transplantation: a minireview. *Transplantation Proceedings*.

[31] Savier E., Granger B., Charlotte F. (2011). Liver preservation with SCOT 15 solution decreases posttransplantation cholestasis compared with university of Wisconsin solution: a retrospective study. *Transplantation Proceedings*.

[32] Dutheil D., Rioja-Pastor I., Tallineau C. (2006). Protective effect of PEG 35,000 Da on renal cells: paradoxical activation of JNK signaling pathway during cold storage. *American Journal of Transplantation*.

[33] Zhong Z., Theruvath T. P., Currin R. T., Waldmeier P. C., Lemasters J. J. (2007). NIM811, a mitochondrial permeability transition inhibitor, prevents mitochondrial depolarization in small-for-size rat liver grafts. *The American Journal of Transplantation*.

[34] Ichimura T., Ito M., Takahashi K., Oyama K., Sakurai K. (2011). Involvement of mitochondrial swelling in cytochrome c release from mitochondria treated with calcium and Alloxan. *Journal of Biophysical Chemistry*.

[35] Kim J.-S., Ohshima S., Pediaditakis P., Lemasters J. J. (2004). Nitric oxide protects rat hepatocytes against reperfusion injury mediated by the mitochondrial permeability transition. *Hepatology*.

[36] Bertuglia S., Veronese F. M., Pasut G. (2006). Polyethylene glycol and a novel developed polyethylene glycol-nitric oxide normalize arteriolar response and oxidative stress in ischemia-reperfusion. *The American Journal of Physiology—Heart and Circulatory Physiology*.

[37] Bouma H. R., Ketelaar M. E., Yard B. A., Ploeg R. J., Henning R. H. (2010). AMP-activated protein kinase as a target for preconditioning in transplantation medicine. *Transplantation*.

[38] Ahn Y.-J., Kim H., Lim H. (2012). AMP-activated protein kinase: implications on ischemic diseases. *BMB Reports*.

[39] Qi D., Young L. H. (2015). AMPK: energy sensor and survival mechanism in the ischemic heart. *Trends in Endocrinology & Metabolism*.

[40] Harada N., Hatano E., Koizumi N. (2004). Akt activation protects rat liver from ischemia/reperfusion injury. *Journal of Surgical Research*.

[41] Zhang R., Zhang L., Manaenko A., Ye Z., Liu W., Sun X. (2014). Helium preconditioning protects mouse liver against ischemia and reperfusion injury through the PI3K/Akt pathway. *Journal of Hepatology*.

[42] Suo L., Kang K., Wang X. (2014). Carvacrol alleviates ischemia reperfusion injury by regulating the PI3K-Akt pathway in rats. *PLoS ONE*.

[43] Yoshinari D., Takeyoshi I., Kobayashi M. (2001). Effects of a p38 mitogen-activated protein kinase inhibitor as an additive to University of Wisconsin solution on reperfusion injury in liver transplantation. *Transplantation*.

[44] Clanachan A. S., Jaswal J. S., Gandhi M. (2003). Effects of inhibition of myocardial extracellular-responsive kinase and P38 mitogen-activated protein kinase on mechanical function of rat hearts after prolonged hypothermic ischemia. *Transplantation*.

[45] Hashimoto N., Takeyoshi I., Yoshinari D. (2002). Effects of a p38 mitogen-activated protein kinase inhibitor as an additive to euro-collins solution on reperfusion injury in canine lung transplantation. *Transplantation*.

[46] Bejaoui M., Pantazi E., Folch-Puy E. (2015). Emerging concepts in liver graft preservation. *World Journal of Gastroenterology*.

